# Radiation-induced lung toxicity – cellular and molecular mechanisms of pathogenesis, management, and literature review

**DOI:** 10.1186/s13014-020-01654-9

**Published:** 2020-09-10

**Authors:** Lukas Käsmann, Alexander Dietrich, Claudia A. Staab-Weijnitz, Farkhad Manapov, Jürgen Behr, Andreas Rimner, Branislav Jeremic, Suresh Senan, Dirk De Ruysscher, Kirsten Lauber, Claus Belka

**Affiliations:** 1 Department of Radiation Oncology, University Hospital, LMU Munich, Marchioninistrasse 15, 81377 Munich, Germany; 2 German Center for Lung Research (DZL), partner site Munich, Munich, Germany; 3 German Cancer Consortium (DKTK), partner site Munich, Munich, Germany; 4 Walther Straub Institute of Pharmacology and Toxicology, Member of the German Center for Lung Research (DZL), Medical Faculty, LMU-Munich, Munich, Germany; 5 Institute of Lung Biology and Disease, Helmholtz Zentrum München, Munich, Germany; 6 Department of Internal Medicine V, LMU Munich, Munich, Germany; 7 Department of Radiation Oncology, Memorial Sloan Kettering Cancer Center, New York, USA; 8 Research Institute of Clinical Medicine, Tbilisi, Georgia; 9 Department of Radiation Oncology, Amsterdam University Medical Centers, Amsterdam, Netherlands; 10 Department of Radiation Oncology (MAASTRO), GROW School for Oncology and Developmental Biology, Maastricht University Medical Centre, Maastricht, the Netherlands

## Abstract

Lung, breast, and esophageal cancer represent three common malignancies with high incidence and mortality worldwide. The management of these tumors critically relies on radiotherapy as a major part of multi-modality care, and treatment-related toxicities, such as radiation-induced pneumonitis and/or lung fibrosis, are important dose limiting factors with direct impact on patient outcomes and quality of life. In this review, we summarize the current understanding of radiation-induced pneumonitis and pulmonary fibrosis, present predictive factors as well as recent diagnostic and therapeutic advances. Novel candidates for molecularly targeted approaches to prevent and/or treat radiation-induced pneumonitis and pulmonary fibrosis are discussed.

## Introduction

Lung, breast, and esophageal cancer are common thoracic malignancies with high cancer-associated mortality [1]. In the majority of cases thoracic radiotherapy represents a central part of multi-modal treatment concepts [2]. Several diagnostic and therapeutic advances, such as PET-imaging [3, 4], improved radiation delivery techniques [5–9], implementation of immunotherapy [10–16], and molecularly targeted therapy [17–19], have led to improved outcome in terms of overall survival, local and distant control as well as quality of life. However, between 10 and 30% of all patients with lung or breast cancer receiving thoracic radiotherapy develop radiation-induced pneumonitis (RIP) as a subacute treatment-associated toxicity, and they are at high risk of developing radiation-induced lung fibrosis (RILF) as late toxicity, although treatment-related death is uncommon [5, 20–24].

Accordingly, lung toxicity remains a crucial dose limiting factor, and dose escalation trials with conventionally fractionated radiotherapy have been limited by severe lung toxicity [25–27]. Due to the development of novel radiotherapy techniques, including intensity modulated radiotherapy (IMRT) [5, 6] and volumetric modulated arc therapy (VMAT) [20], and radiation qualities, such as and proton therapy [28], radiation exposure of normal lung tissue can be significantly reduced. Consequently, the occurrence of RIP grade ≥ 2 in the treatment of lung cancer has gradually decreased from 30 to 47% using 2D-radiotherapy [29], to 30–35% with 3D-radiotherapy [30, 31], 29–32% with IMRT [31, 32], 24–29% with VMAT [32, 33], and < 5% with proton therapy [28, 34]. The radiation delivery technique is also of importance for the development of RIP and RILF. Different fractionation regimens, such as classically fractionated radiotherapy with 2 Gy per fraction for the treatment of rather large tumor volumes, and high precision radiation delivery techniques for the treatment of smaller volumes, such as stereotactic body radiotherapy (SBRT) or stereotactic ablative radiotherapy (SABR), are associated with different risk profiles of RIP/RILF arising from differences in the delivered doses and target volumes. In addition, single- versus multi-fraction course SBRT/SABR regimens and the localization of the tumor (central versus peripheral) impact radiation-induced lung toxicity [35–37]. Central tumors treated with SBRT often receive more conservative dose fractionation regimens (e.g. SBRT with 3–8 fractions) compared to peripheral tumors resulting in less treatment-related toxicity but comparable outcome [37–39]. High dose single-fraction lung SBRT (e.g. ≥ 30 Gy) may be associated with increased toxicity [40, 41]. However, several studies reported low rates of ≥ grade III side effects in selected patient cohorts [35, 42].

This review summarizes the current understanding of the cellular and molecular mechanisms underlying the pathogenesis of RIP and RILF. We present predictive factors and the current standards of diagnostic and therapeutic management. Finally, we discuss novel candidates for molecularly targeted approaches to prevent and/or treat RIP and RILF.

### Diagnosis of RIP and RILF

The diagnosis of RIP and RILF is based on clinical presentation and may be supported by associated imaging findings. Various grading scales are used (see Tables 1 and 2). In clinical practice, Radiation Therapy Oncology Group (RTOG) criteria and the Common Terminology Criteria for Adverse Events (CTCAE) are the ones most widely used [43, 44]. However, the majority of all patients will not show any clinical symptoms. Upon conventional thoracic radiotherapy, RIP occurs 1 and 6 months after treatment, typically within 3 months following completion of irradiation. Clinical symptoms include persistent, dry and non-productive coughing, dyspnea (on physical exertion or at rest), low-grade fever, pleuritic pain, and chest discomfort [45]. To date, no standard laboratory test can unequivocally identify RIP. Most patients exhibit normal levels of C-reactive protein (CRP) and diagnostic differentiation from bacterial pneumonia remains challenging [46]. Nevertheless the performance of bronchial lavage sampling with subsequent cytology and immunomonitoring analyses for differential diagnosis of RIP from infectious lung disease is currently under investigation [47].

**Table 1:** Overview about grading scales for radiation-induced pneumonitis

Grading scale	Grade 1	Grade 2	Grade 3	Grade 4	Grade 5
CTCAE v5.0	Asymptomatic; clinical or diagnostic observations only; intervention not indicated	Symptomatic; medical intervention indicated; limiting instrumental ADL	Severe symptoms; limiting self care ADL; oxygen indicated	Life-threatening respiratory compromise; urgent intervention indicated (e.g., tracheotomy or intubation)	Death
RTOG	Asymptomatic or mild symptoms (dry cough); slight radiographic appearances	Moderate symptomatic pneumonitis (severe cough); low grade fever; patchy radiographic appearances	Severe symptomatic pneumonitis; dense radiographic changes	Severe respiratory insufficiency/ Continuous O2/ Assisted ventilation	Death
LENT-SOMA (EORTC)	Asymptomatic or mild symptoms; slight imaging changes	Moderate symptoms; moderate imaging changes	Severe symptoms; increased density imaging changes	Severe symptoms requiring continuous O2 or assisted ventilation	Death

*CTCAE v5.0* Common terminology criteria for adverse events, version 5.0, *RTOG* Radiation Therapy Oncology Group, *EORTC* European Organization for Research and Treatment of Cancer, *LENT-SOMA* Late effects in normal tissue-subjective objective management analysis

**Table 2:** Overview about grading scales for radiation-induced lung fibrosis

Grading scale	Grade 1	Grade 2	Grade 3	Grade 4	Grade 5
CTCAE v5.0	Radiologic pulmonary fibrosis < 25% of lung volume associated with hypoxia	Evidence of pulmonary hypertension; radiographic pulmonary fibrosis 25–50% associated with hypoxia	Severe hypoxia; evidence of right-sided heart failure; radiographic pulmonary fibrosis > 50–75%	Life-threatening consequences (e.g., hemodynamic/pulmonary complications); intubation with ventilatory support indicated; radiographic pulmonary fibrosis > 75% with severe honeycombing	Death
RTOG	Asymptomatic or mild symptoms (dry cough); slight radiographic appearances	Moderate symptomatic fibrosis (severe cough); low grade fever; patchy radiographic appearances	Severe symptomatic fibrosis; dense radiographic changes	Severe respiratory insufficiency/ Continuous O2/ Assisted ventilation	Death
LENT-SOMA (EORTC)	Asymptomatic or mild symptoms; radiological abnormalities; 10–25% reduction of respiration volume and/or diffusion capacity	Moderate symptoms; patchy dense abnormalities in imaging; > 25–50% reduction of respiration volume and/or diffusion capacity	Severe symptoms; dense confluent radiographic changes limited to irradation field; > 50–75% reduction of respiration volume and/or diffusion capacity	Severe symptoms requiring continuous O2 or assisted ventilation; dense fibrosis, severe scarring & major retraction of normal lung; > 75% reduction of respiration volume and/or diffusion capacity	Death

*CTCAE v5.0* Common terminology criteria for adverse events, version 5.0, *RTOG* Radiation Therapy Oncology Group, *EORTC* European Organization for Research and Treatment of Cancer, *LENT-SOMA* Late effects in normal tissue-subjective objective management analysis

The benefit of lung function tests for determining the grade of RIP, such as spirometry with lung diffusion capacity test, remains unclear. Several studies investigated changes in lung function after thoracic radiotherapy, and the extent of change in diffusing capacity of lung for carbon monoxide (DLCO) upon radiotherapy of non-small cell lung cancer (NSCLC) patients was reported to be associated with the RIP grade [48, 49]. However, no national or international consensus has yet been established.

Imaging of RIP upon conventional radiotherapy may present with non-specific chest X-ray changes which typically are confined to the irradiated field, with airspace opacities being most common [50]. Pleural effusions or atelectasis may be associated as well. The preferred imaging modality to detect RIP is chest computed tomography (CT), preferably high-resolution computed tomography (HRCT). Chest CTs provide more detailed information about parenchymal changes and often reveal alterations that are localized to the irradiated field, rendering the diagnosis of RIP for clinicians rather obvious [51]. The radiological characteristics of RIP change over time. In the initial phase they include ground-glass opacities and/or airspace consolidation [52]. In some cases, a small ipsilateral pleural effusion may occur in the first 6 months after thoracic irradiation and may persist for several months. In the later phase of RIP after conventional radiotherapy, septal wall thickening may occur with the alveolar opacities producing a “crazy paving” pattern [53]. Upon SABR, radiographic changes will occur in most of the patients within 6 months and can be described as diffuse consolidation (> 20%), patchy consolidation (> 20%), and diffuse or patchy ground glass opacities (> 5%) (see Table 3) [54, 55]. In contrast to conventional radiotherapy, these changes do usually not occur before 2–3 months after completion of treatment – presumably due to the relevantly shorter treatment course. [18F]fluoro-2-deoxy-2-D-glucose positron emission tomography combined with computed tomography (18F-FDG PET/CT) does not contribute to confirming a RIP diagnosis [56]. Inflammatory processes usually demonstrate an increased metabolic activity and are common after thoracic radiotherapy, causing significant confusion when PET/CT is used in the first 6 months after irradiation. However, only the minority of these patients develop clinical RIP.

**Table 3:** Overview of radiographic changes after completion of conventionally fractionated radiotherapy compared to stereotactic ablative radiotherapy (SABR) of the thorax

	Conventionally fractionated radiotherapy	Stereotactic ablative radiotherapy (SABR)
Radiographic changes within 6 months after completion of radiotherapy	•consolidation conform to irradiation field •diffuse ground glass opacities and/or airspace consolidation •nodule-like pattern •atelectasis •(ipsilateral) pleural effusion	•diffuse and/or patchy consolidation •diffuse and/or patchy ground glass opacities
Radiographic changes after 6 months following completion of radiotherapy	•scar-like fibrosis > conventional pattern > mass-like fibrosis •volume loss •linear scarring/restriction to radiation fields •chronic consolidation ± air-bronchograms •bronchiectasis •pleural thickening •hilar vascular displacement •mediastinal shift •(ipsilateral) pleural effusion	•modified conventional pattern > scar-like fibrosis > mass-like fibrosis •chronic consolidation •volume loss •bronchiectasis

RILF is typically observed between 6 and 12 months after thoracic radiotherapy and can continuously progress for several years. Several grading scales have been established to categorize RILF (see Table 2). Nearly all patients show (radiographic) signs of RILF following thoracic irradiation [50]. However, the majority of patients with RILF remain asymptomatic, and clinical manifestations are mostly linked to established comorbidities, such as pre-existing lung or heart disorders. Symptoms include dyspnea (upon physical exertion or at rest), persistent and dry coughing, fatigue, and weight loss [45]. Radiographic pulmonary changes are usually observed in the irradiated field but can occur in the rest of the lung as well [57].

Chest X-ray imaging can show volume loss and architectural distortion [56]. In some cases, a mediastinal shift and traction bronchiectasis can be found. Compared to previous chest X-ray scans, progression from RIP increasingly becoming more reticular or linear is typical for RILF. Again, HRCT imaging can better delineate parenchymal changes as compared to chest X-ray imaging, including volume loss, linear scarring, and traction bronchiectasis [58]. Chronic consolidation is often found together with air-bronchograms usually exhibiting a non-anatomical distribution. Upon SABR the most frequent late radiographic changes are characterized by consolidation, volume loss, and bronchiectasis in a so called “modified conventional pattern”. Previously, straight lines or edges were typically reported as pulmonary changes following conventional 2D- or 3D-radiotherapy. Yet, with current delivery techniques, such as IMRT, VMAT, or SABR, these changes have become rather uncommon, and/or may be more difficult to appreciate without information on the beam configurations used.

A special and very rare form of lung toxicity upon RT for breast cancer is radiation-induced bronchiolitis obliterans organizing pneumonia (BOOP, < 2% of cases) which may develop several months after RT, commonly with longer latency time compared to RIP [59]. Clinical presentation and radiographic changes are similar to RIP. In contrast to RIP which largely remains limited to the irradiated fields, BOOP is frequently found also in the non-irradiated lung with diffuse patterns and may show patchy alveolar infiltrations ± air bronchograms and consolidations [60].

The severity of RILF can be radiologically measured with the help of semi-quantitative grading (1–5 points) using radiographic features (see Table 3). “Scar-like” patterns as characterized by streaky opacities in the tumor region are usually associated with less severe RILF due to the mild loss of volume [56]. Conversely, “mass-like” patterns as depicted by focal consolidation and/or ground glass opacification in the tumor region typically with air bronchograms and/or traction bronchiectasis represent rather severe forms of RILF [56]. Upon SABR, “mass-like” fibrosis has been observed more frequently (in up to 14% of cases) and challenges the diagnosis of local recurrence [54]. In contrast to the equivocal value of 18F-FDG-PET/CT for RIP diagnosis, it can be helpful in differentiating pulmonary fibrosis of radiation-induced origin from recurrent malignancy [56].

### Cellular and molecular mechanisms and pathogenesis of RIP and RILF

**Fig. 1:**
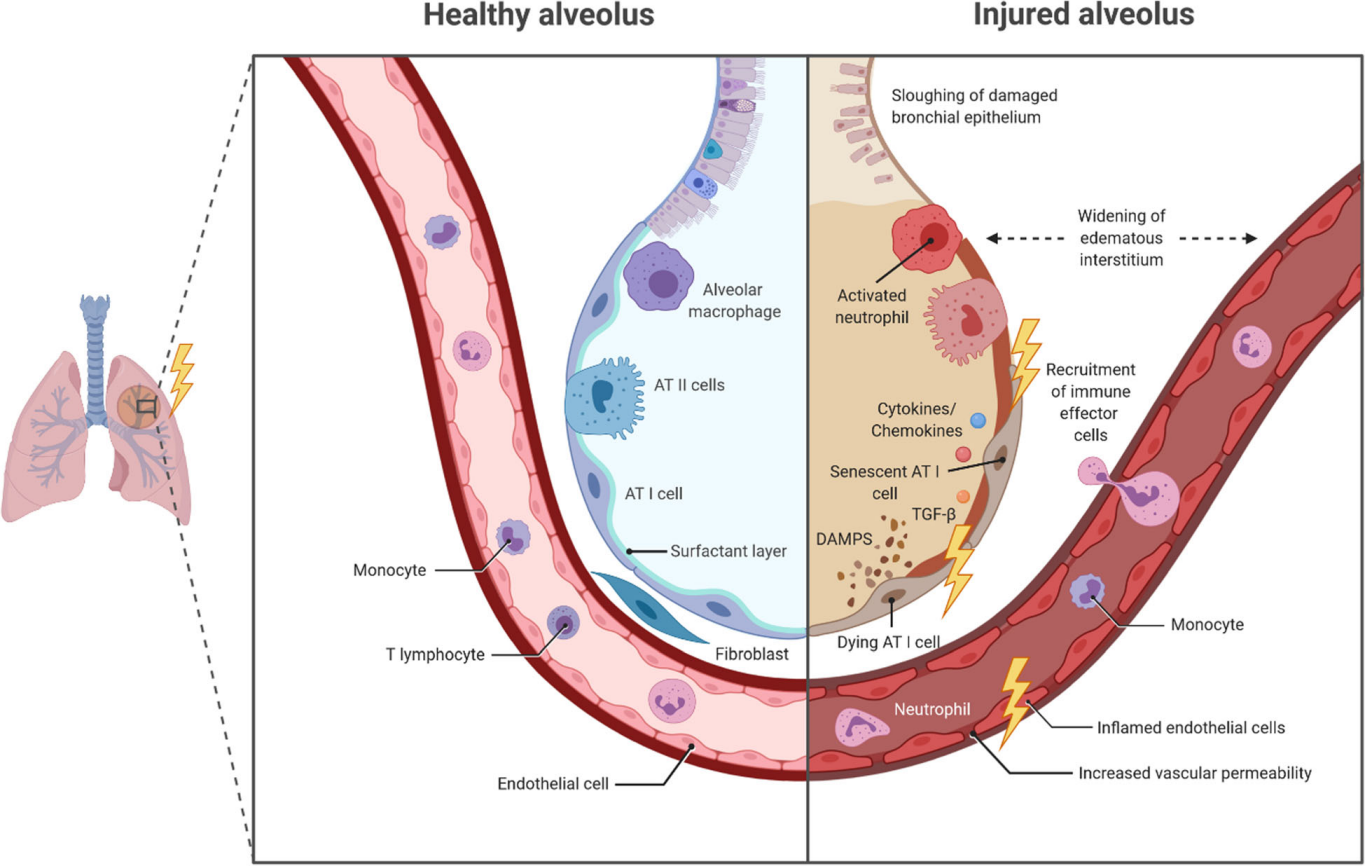
Overview about radiation-induced acute and subacute alveolar changes. AT I cell: alveolar type I cell; AT II cell: alveolar type II cell; DAMP: damage-associated molecular pattern; TGF-β: transforming growth factor β

**Fig. 2:**
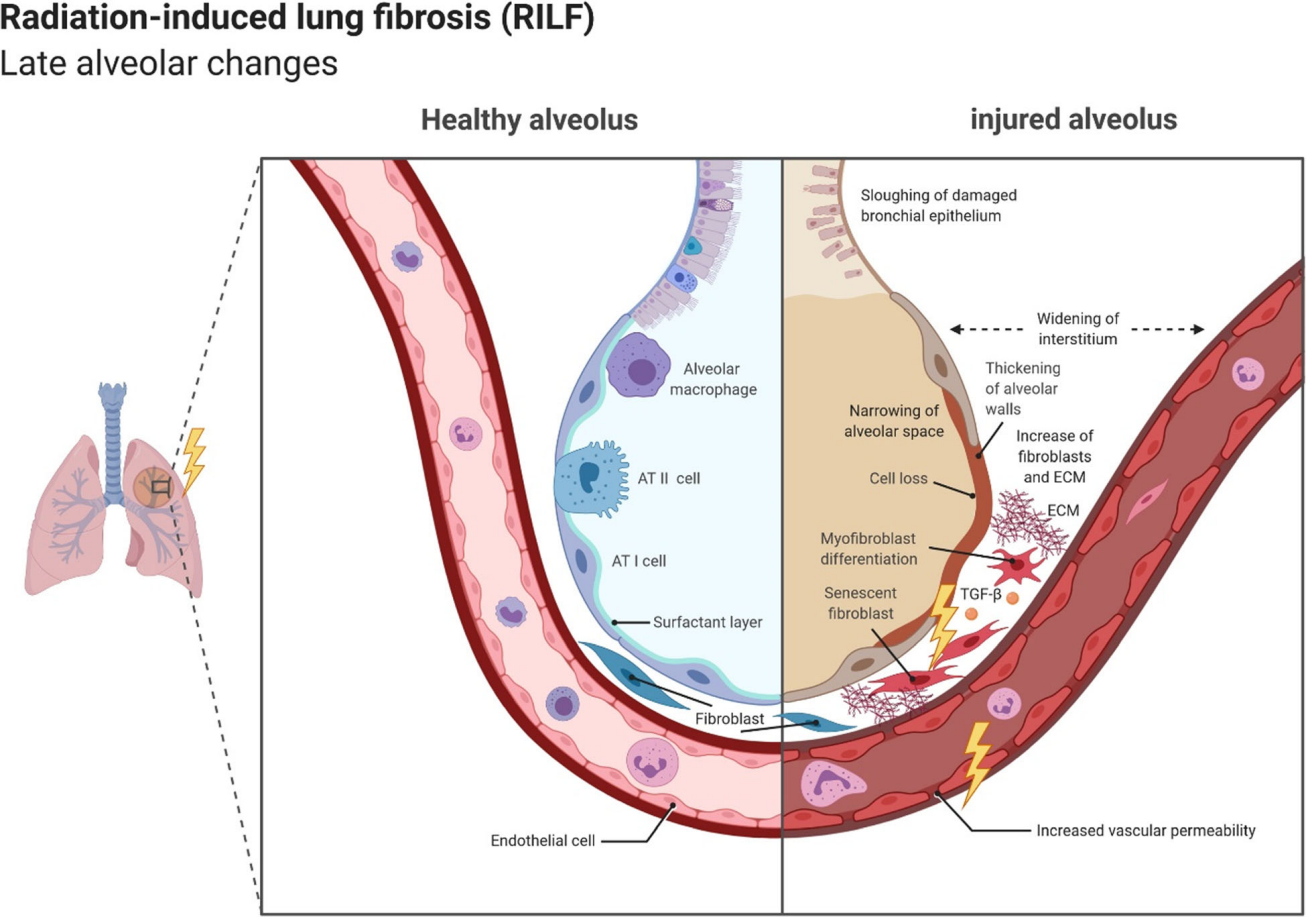
Overview about radiation-induced late alveolar changes. AT I cell: alveolar type I cell; AT II cell: alveolar type II cell; ECM: extracellular matrix; TGF-β: transforming growth factor β

The alveolar tissue of the lung is relatively sensitive to ionizing radiation [61, 62]. Therefore, RIP and RILF are major dose limiting adverse effects interfering with the radiotherapeutic success in the treatment course of thoracic malignancies [63–66]. The pathogenesis of RIP and RILF is a complex multi-step process involving several resident cells of the lung as well as recruited immune cells and is initiated and perpetuated via pleiotropic inter- and intracellular communication and signaling events [67–69]. According to the current understanding, an overwhelming cascade of damage-associated molecular patterns (DAMPs), pro-inflammatory cytokines, and chemokines released by dying and/or senescent epithelial cells, endothelial cells, and activated immune cells essentially contribute to the development of RIP and RILF (see Figs. 1 and 2) [66, 70].

Radiation-induced lung toxicity can be divided into three phases: Acute, subacute, and late radiation toxicity. In the acute phase, occurring minutes to days after irradiation, repair of radiation-induced DNA damage takes place in the lung tissue. This includes base modifications, single and double strand breaks of varying complexity, DNA crosslinks, and bulky lesions which arise from direct ionization events or are indirectly mediated by free reactive oxygen species (ROS), respectively [71]. Acute radiation-induced toxicity appears to primarily involve alveolar type I (AT I) and II (AT II) epithelial cells, and endothelial cells [62]. Whereas most tumor cells preferentially undergo necrotic forms of cell death upon radiation at clinically relevant doses, normal tissue epithelial cells and endothelial cells predominantly show phenotypes of cellular senescence [66, 72]. Intriguingly, radiation-induced senescence is accompanied by an altered gene expression profile and the release of several pro-inflammatory cytokines and chemokines, constituting the senescence-associated secretory phenotype (SASP) [73]. Major representatives of SASP cytokines include transforming growth factor-β (TGF-β), platelet-derived growth factor (PDGF), interleukins (IL) -1, − 6, and − 8 as well as ligands of the CXCR1/2 and CCR2/5 chemokine receptors. These mediators enforce cellular senescence in parenchymal cells, stimulate endothelial cell activation, and contribute to the recruitment and activation of immune cells [68]. Moreover, DNA damage-induced senescence and death of AT I and AT II cells result in a loss of barrier function and reduced surfactant production, decreased surface tension, and possible atelectasis due to the lack of surfactant eventually leading to interstitial edema, exudation of proteins into the alveolar space, and further reduction of the alveolar septa [74–77]. The microvascular system of the lung tissue and particularly endothelial cells are also affected by radiotherapy, both directly via DNA damage-induced senescence and indirectly via released DAMPs and SASP cytokines giving rise to increased vascular permeability and decreased vascular integrity further amplifying the damage of AT I and AT II cells (see Fig. 1) [76]. Ultimately, the affected lung tissue will develop sterile alveolitis with further infiltration of immune cells.

The subacute phase, which can last for several months, is defined by the recruitment of several effector cells of the innate and adaptive immune system (neutrophils, monocytes, macrophages, and lymphocytes) and the concomitant release of pro-inflammatory cytokines which trigger extensive tissue remodeling of the lung. Immune cell infiltration into the injured lung is facilitated by increased vascular permeability, augmented expression of adhesion molecules (e.g. intercellular adhesion molecule 1 (ICAM-1) and platelet endothelial cell adhesion molecule 1 (PECAM-1) on activated endothelial cells, and release and deposition of chemokines [78]. The initial recruitment of neutrophils is followed by monocytes, macrophages, and lymphocytes (see Fig. 1) [79, 80], and immune-cell derived cytokines, including tumor necrosis factor (TNF), TGF-β, IL-2, IL-3, IL-4, IL-6, IL-7, and IL-8, enforce the activation and proliferation of fibroblasts [81–84]. For a more detailed assessment of the contribution of distinct immune cell subsets, the interested reader is referred to Wirsdörfer et al. [85], Kainthola et al. [86] and McKelvey et al. [87].

Apart from the described immune-mediated tissue remodeling events, hypoxia has been reported to contribute to the onset and the perpetuation of RIP and RILF [76]. Radiation-induced hypoxia occurs several days after thoracic radiotherapy and has been reported to increase over time in different animal models [76, 88]. Importantly, hypoxia-induced downstream signaling leads to upregulation of TGF-β, enhanced collagen synthesis, and changes in the lung architecture (see Fig. 2). In summary, all these events contribute to the development and establishment of RIP which represents the acute, but reversible scenario of radiation-induced lung toxicity. Of note, elevated serum levels of TGF-β are associated with increased risk of RIP [89].

The late phase of radiation-induced lung injury can be defined by the irreversible rearrangement of lung architecture which occurs several months following thoracic radiotherapy [58]. Again, TGF-β produced by activated immune cells as well as by AT I/II cells and fibroblasts, appears to be a key player due to its profibrotic functions (see Fig. 2) [89, 90]. TGF-β acts by binding to two serine/threonine kinase receptors, namely TGF-β type I and type II receptors, resulting in the activation of multiple signaling cascades, including the small mother against decapentaplegic (SMAD) 2/3, mitogen-activated protein kinase (MAPK) and extracellular signal–regulated kinase (ERK) signaling pathways [77, 91–93]. Activated Smad2/3 forms complexes with Smad4, subsequently translocating to the nucleus and regulating the expression of genes associated with fibroblast proliferation, migration, and collagen synthesis in the lung tissue [77, 91]. TGF-β stimulates the expression of fibrillar collagens (type I, III and type V) and fibronectin by fibroblasts in the interstitial space resulting in stiffening of the alveolar area and reduction of gas exchange [94–96]. Additionally, overexpression of TGF-β in experimental models of fibrosis was observed to be accompanied by upregulation of protease inhibitors, such as tissue inhibitor of metalloproteinases (TIMP) and plasminogen activator inhibitor-1 (PAI-1), along with an excessive accumulation of matrix proteins and collagens [97, 98]. TGF-β stimulates the differentiation of fibroblasts into myofibroblasts which comes along with induction of α-smooth muscle actin (α-SMA) and increased contractility [99]. Thus, lung architecture remodeling culminates in increasing stiffness and thickening of the lung parenchyma due to the overproduction of extracellular matrix proteins, and the alveolar space is severely reduced [100]. These architectural changes in the lung and the expansion of irreversibly fibrotic regions during the late phase after thoracic radiotherapy are apparent in chest CTs as pulmonary fibrosis.

### Predictors of RIP

RIP occurs in the subacute phase after radiotherapy and is mainly characterized by increased infiltration of immune effector cells, such as neutrophils, monocytes, and macrophages, and the release of pro-inflammatory cytokines and chemokines. In order to prevent the development of RIP and RILF in the radiotherapeutic routine, several risk factors have been identified. The predictors of RIP can be patient-, disease-, and/or treatment-related.

#### Patient-related risk factors of RIP

Several patient-related characteristics, such as age, sex, performance status, smoking status, and comorbidities, have been suggested as risk factors for RIP. In a meta-analysis of 31 independent studies with patients of different thoracic malignancies (lung, breast, and esophageal cancer), older age (odds ratio (OR) = 1.7, *p* < 0.0001) and pre-existing comorbidities (OR = 2.3, *p* = 0.007) were identified as potential risk factors for the development of RIP [101]. In contrast, a subsequent study with 576 patients identified no significant differences in the incidence of grade ≥ 3 RIP between patients ≤60 and > 60 years (*p* = 0.943) [102], and other studies also failed to find significant associations between increasing age and the occurrence of RIP [102–104]. In summary, age should be considered as a relevant risk factor for RIP, but lung comorbidities and radiotherapy features may be more important risk factors compared to chronological age alone.

The role of the patients’ sex remains controversial. In average, women appear to have smaller tumor volumes and have more often a non-smoking history compared to men [105]. Therefore, their pre-radiotherapy lung capacity (FEV1, DLCO) is usually better than the one of male patients.

Pre-existing lung disease, such as chronic obstructive pulmonary disease (COPD) or interstitial lung disease (ILD), can confound the diagnosis of RIP and occurs quite frequently in lung cancer patients. The predictive role of COPD appears controversial. While patients with extensive emphysema experience RIP in more than 50%, and several studies suggest an increased risk of RIP in patients with underlying COPD [106, 107], other reports do not confirm these observations [102, 108]. Patients with pre-existing ILD seem to be more susceptible to RIP and are at markedly increased risk of radiation-induced toxicity [109, 110]. A retrospective analysis of 504 patients undergoing thoracic SABR reported grade ≥ 3 RIP in 32% and grade 5 pneumonitis in 21% of all ILD patients compared to a general risk of less than 10% of grade ≥ III RIP after SBRT [111–113]. Further studies observed an SABR-related mortality rate of 16%, and it was recommended to reduce the radiation dose for patients with pre-existing ILD in order to prevent radiation-induced lung toxicity [114]. Accordingly, stricter than normal dose constraints may need to be applied in these cases [110, 115], and careful weighing of the risks and benefits for each individual patient is critical in this population at high risk for severe toxicity. Informed consent should include a clear description of the risks. Alternative treatment options, including close observation, should be explored and considered [109]. Interstitial lung abnormalities (ILAs) are defined as precursor stages of idiopathic pulmonary fibrosis and show similar, but less severe radiological changes compared to ILD [116]. Although ILAs mostly remain asymptomatic or subclinical, they are frequently observed in lung cancer screening trials and need particular attention [117]. The ILA classifying radiographic changes include non-fibrotic (ground glass opacification, areas of consolidation, mosaic attenuation) as well as fibrotic features (ground glass opacification with reticulation, honeycombing). Importantly, patients with ILAs show lower exercise tolerance, a restrictive pattern in lung function tests, higher risk of developing clinically significant ILD, and an increased overall mortality [118]. Along these lines, patients with pre-existing ILAs also seem to be more susceptible to radiation-induced toxicity [109, 110]. Therefore, physicians should perform a comprehensive risk assessment, including clinical (prior symptoms, diagnosis, lung function with DLCO) and image-based evaluation, and the radiotherapeutic treatment of patients with ILA should be carefully discussed – preferentially in a process of shared decision-making. Ongoing trials, such as ASPIRE-ILD phase II study (NCT03485378), are currently prospectively investigating the safety and efficacy of SBRT in patients with inoperable early stage NSCLC with pre-existing ILD and ILA [119].

#### Disease-related risk factors of RIP

Disease-related factors of RIP include the tumor location and the tumor volume. The location of the tumor was reported to be associated with the risk of RIP in several studies and meta-analyses identifying patients with tumors in the middle or lower lobes to be at higher risk [101, 103, 120]. A significantly elevated risk of RIP was described for patients with tumors in the inferior part of the lung [103]. The increased risk of RIP may reflect differences in radiosensitivity between different regions of the lung. The caudal part of the lung contains more lung volume and shows stronger movements compared to the cranial part – especially in patients with emphysema.

In addition, increasing tumor volumes seem to be associated with higher probabilities to develop RIP [121–124]. Accordingly, treatment volume planning, motion management, and delivery verification strategies are critical. Nevertheless, there is currently no consensus in the literature on the reporting of cut-offs as well as on the used radiation delivery techniques. Moreover, tumor volumes can be described by different measures which are inconsistently used, including gross tumor volume (GTV), clinical target volume (CTV), planning target volume (PTV) ± involved lymph node volume, and lung volume minus GTV, CTV, or PTV, respectively. Interestingly, the irradiated lung volume was not significantly associated with radiation-induced BOOP after radiotherapy for breast cancer [60].

Apart from the tumor volume and its location, its proximity to the heart and – in consequence – the radiation dose delivered to the heart impacts the risk of RIP and RILF [125–127]. Importantly, the dosimetric values of the heart are not simply surrogate markers for dosimetric lung parameters [127]. The underlying mechanisms have not been understood yet, but dose constraints to the heart need to be critically considered to prevent RIP and RILF.

In contrast, the tumor stage has not been confirmed as a risk factor for RIP [46, 102, 128]. Hence, tumor volume rather than tumor stage should be considered as a relevant risk factor for RIP, but clear cut-off values remain to be defined for both conventional and SABR populations.

#### Treatment-related risk factors of RIP

Treatment of thoracic malignancies involves radiotherapy, surgery, and various systemic therapies. As a result, different treatment modalities are accompanied by different risks for the development of RIP. Several studies reported that previous surgery leads to a higher risk of RIP [126, 129]. However, in a meta-analysis including 6 studies with 800 patients, surgery was not confirmed as a risk factor for RIP [101]. The extent of resection and differences in postoperative treatments may represent confounding factors and thus should be analyzed in greater detail.

Systemic treatment options include several different agents, combinations, and that affect radiation-induced lung toxicity [130]. Compared to other anticancer drugs, paclitaxel-based chemotherapy has been described to be associated with higher risks of RIP [124, 131–133]. Additionally, a meta-analysis found that sequential rather than concurrent chemotherapy (OR = 1.6, *p* = 0.01) seems to increase the RIP risk. Yet, treatment intensity and patient selection may confound these findings and thus need to be considered [101]. Conflicting results were reported in a different meta-analysis including 1205 patients from seven randomized clinical trials which showed no significant differences between concomitant and sequential chemotherapy for grade ≥ 3 acute pulmonary toxicity (relative risk (RR): 0.69; 95% CI: 0.42 to 1.12; *p* = 0.13) [134].

Parameters extracted from dose-volume histograms may offer the most resilient predictors of radiation-induced toxicity. In the literature, the mean lung dose (MLD) and the lung volume receiving 20 Gy (V20) are the most frequently and robustly reported risk factors [124, 135]. It is recommended to limit V20 to ≤30–35%, and MLD to ≤20–23 Gy in normofractionated radiotherapy to limit the risk of RIP to ≤20% in patients with NSCLC [124]. Hypofractionated radiotherapy with single doses of ≥2.5 Gy is associated with higher rates of RIP [124, 136]. For SBRT, V20 > 10% and MLD > 6 Gy were associated with higher risk of grade 2–4 RIP [137–139]. Apart from these established dose constraints, the concept of the “critical volume” has been increasingly used [140]. According to this concept, a minimum of approximately one-third of the total native lung volume (with connection to the body via a functional hilum) needs to be spared from the threshold dose in order to maintain the basic organ function. Several protocols defining the critical lung volume have been published, ranging from 1000 to 1500 cm^3^ [140–142]. Future studies are needed to provide additional guidance for physicians and to assess the performance of the critical volume concept with regards to preventing radiation-induced toxicity.

With the clinical implementation of immunotherapeutic protocols, the impact of immune checkpoint inhibition (ICI) on the development of RIP needs to be examined and is currently under investigation [143, 144]. Programmed cell death 1 (PD-1)/ Programmed cell death 1 ligand 1 (PD-L1) inhibition alone can cause immune-mediated pneumonitis in less than 5% [145]. Furthermore, radiation recalls several months after thoracic radiotherapy while ICI is still being administered have been described in some cases [144, 146]. The first systematic retro- and prospective studies have shown acceptable toxicity of sequential and concurrent radio-immunotherapy [147–150]. However, the risk of RIP and immune-mediated pneumonitis may still be underestimated [144, 151]. Unfortunately, predictive biomarkers and/or patient- or disease-related characteristics that can identify patients with elevated risk of RIP with ICI treatment are currently not available [152], but several ongoing studies are investigating these multi-modal treatment approaches and aim at establishing such biomarkers (NCT03519971 (PACIFIC-2), NCT04245514 (SAKK 16/18), NCT03801902 (NRG-LU004), NCT03217071). For the time being, careful monitoring of radiation and/or immune-mediated pneumonitis and appropriate treatment management strategies with the aim of reducing risk and/or enabling early symptom detection are needed [153].

### Prevention of radiation-induced lung injury

Although distinct improvements in radiation treatment planning and delivery techniques (IMRT, VMAT) allow sparing the healthy tissue while escalating the dose administered to the tumor, RIP and RILF remain dose limiting factors of thoracic radiotherapy which strongly affect the therapeutic outcome and quality of life. In order to improve outcome in patients with locally advanced stages of thoracic cancer, multi-modal treatments combining radio-, chemo- and/or immunotherapy are increasingly being employed and often represent the standard of care [13, 14, 154]. Besides technical advances to reduce radiation-induced toxicity, such as the implementation of IMRT and VMAT, no evidence-based pharmacological intervention has been found so far. Several agents are currently under investigation to prevent and/or treat RIP and RILF, namely protectors, modifiers, and mitigators of radiation-induced lung toxicity. Diverse mechanisms of action have been suggested. As such, radiation protectors would be given before radiotherapy, mitigators would be administered during or immediately after irradiation but before the occurrence of radiation-induced toxicity, and modifiers of radiation-induced lung toxicity would be employed after the appearance of RIP or RILF in order to attenuate progression or to reverse the damage. However, the best strategy seems to be investigating novel radiation delivery techniques (image-guided radiotherapy (IGRT), magnetic resonance (MR)-guided radiotherapy) and radiation qualities (proton, particle therapy) combined with promising pharmacological intervention in order to obtain optimal results.

ACE (angiotensin-converting enzyme) inhibitors and angiotensin-II receptor subtype 1 (AT-1) antagonists have been shown to be helpful in mitigating radiation-induced damage by targeting inflammatory and fibrogenic pathways in preclinical model systems [90, 155, 156]. Angiotensin-II stimulates TGF-β expression via upregulation of toll-like receptor 4 (TLR4) [157] and α-SMA via mechanisms involving serum response factor (SRF) [158]. Accordingly, AT-1 receptor antagonists may counteract these effects. The ACE inhibitor enalaprilat as the active metabolite of enalapril has been reported to attenuate levels of TGF-β, vascular remodeling, and subsequent lung fibrosis [156]. Similarly, the application of captopril was associated with a reduction in pulmonary complication-associated mortality after total body irradiation in a randomized controlled trial [159]. Despite of the strong preclinical evidence, ACE inhibitors and AT-1 receptor antagonists need to be investigated further in prospective trials.

Amifostine is traditionally used to attenuate accumulating renal toxicity and/or xerostomia during anti-cancer chemo (radio)therapy. Several clinical trials incorporating amifostine reported a particularly low rate of clinically apparent pneumonitis upon thoracic chemoradiotherapy for lung cancer patients [160–165]. However, major methodological limitations, including lacking predefinition of time, type of evaluation, lacking inclusion of established risk factors (radiotherapy doses and volumes), and missing control groups limit the informative value of these studies. In the so far largest clinical trial on amifostine only “late lung toxicity” was evaluated [166], and none of the mentioned studies found a reduced rate of clinically and/or radiologically detectable subacute or late lung toxicity upon administration of amifostine [167]. In contrast to radioprotective effects on normal tissues, tumor-protective effects of amifostine can be largely ruled out [167, 168]. However, amifostine can cause adverse effects ranging from nausea and hypotension to myocardial infarction and a poor tolerability (especially when administered intravenously), thus limiting its clinical use.

Prophylactic use of inhalative corticosteroids has been suggested to prevent radiation-induced lung toxicity. However, despite encouraging preclinical results, clinical trials failed to show efficacy of inhalative corticosteroids in the prevention of RIP and RILF [169, 170]. Symptomatic RIP grade 2 patients were successfully treated with inhaled steroids, such as beclomethason [170]. Nevertheless, not all patients may respond to inhaled treatment, and treatment intensification could be necessary with close clinical observation. In contrast to the oral application with a high first pass effect, inhaled application of corticosteroids is accompanied by lower risks of systemic side effects, such as weight gain, hyperglycemia, and sleep disturbances, and thus should be investigated in larger trials.

Pentoxifylline is a phosphodiesterase inhibitor which downregulates the production of pro-inflammatory cytokines, particularly TNF. In preclinical studies, administration of pentoxifylline prior to whole thorax irradiation has been reported to reduce TNF mRNA and protein levels [171]. Furthermore, pentoxifylline-mediated phosphodiesterase inhibition results in reduced leukocyte adherence to endothelial cells, less platelet aggregation, and dilatation of capillaries. In a small placebo-controlled phase II study, pentoxifylline reduced the occurrence of high grade pneumonitis and decreased lung function loss after 3 and 6 months [172] confirming earlier results [173, 174]. However, the small number of included patients, heterogeneous treatment and follow-up monitoring as well as the different primary endpoints of the studies need to be considered, and further randomized controlled trials are warranted.

Mechanistically, TGF-β is a central player in the development of both RIP and RILF. Thus, inhibition of TGF-β and/or its downstream signaling cascades represents an attractive strategy to prevent radiation-induced lung toxicity. Several in vivo studies described reduced inflammation and lung fibrosis upon TGF-β receptor inhibition with LY2109761, a TGF-β receptor I/II kinase inhibitor which interferes with SMAD1/2 phosphorylation, attenuates TGF-β signaling, and suppresses production of the pro-inflammatory cytokines IL-6, IL-7R, and IL-8 [175, 176]. LY2157299 (galunisertib) more specifically inhibits TGF-β receptor I and has already convinced in phase II clinical trials for idiopathic pulmonary fibrosis (IPF) with manageable toxicity [177]. Its relevance for the prevention of RILF remains to be evaluated. Pirfenidone is an anti-fibrotic agent with approval for the treatment of idiopathic pulmonary fibrosis (IPF) that also counteracts TGF-β signaling by downregulating pro-fibrotic cytokines, attenuating lung fibroblast proliferation, and decreasing extracellular matrix deposition [178–181]. Several ongoing or unpublished trials currently investigate pirfenidone for its prophylactic performance in radiation-induced lung toxicity (NCT02296281, NCT00020631).

Platelet-derived growth factor (PDGF) is another cytokine involved in the development of RILF via its engagement in downstream signaling of fibrotic cytokines, such as TGF-β, IL-1, and TNF [176]. Along these lines, the preventive potential of several PDGF receptor inhibitors has been investigated in the context of radiation-induced lung toxicity in vitro and in vivo [182, 183]. Collectively, the findings suggest that the development of lung fibrosis can be inhibited by perturbing fibrotic signaling events and that this strategy is more promising than interfering with inflammation [183]. However, in clinical trials for idiopathic pulmonary fibrosis (IPF), PDGF inhibitors, such as imatinib, failed to prolong survival and/or improve lung function [184] – in contrast to nintedanib which appears safe and slowed down IPF progression considerably [185, 186]. Clinical performance of PDGF inhibitors for the prevention of radiation-induced lung toxicity is currently being trialed (NCT02496585, NCT02452463).

Connective tissue growth factor (CTGF) is a further potential target for the prevention of RILF that was adopted from trials on IPF. It is a matricellular protein involved in tissue remodeling, myofibroblast differentiation, adhesion, and angiogenesis. In vivo experiments demonstrated that CTGF inhibition can attenuate the development of radiation-induced lung fibrosis and even to revert the fibrotic processes in a therapeutic setting [187]. Moreover, FG-3019 (pamrevlumab), a neutralizing antibody designed against CTGF, appears to be more potent than pirfenidone or nintedanib (PDGFR/VEGFR/FGFR inhibitor) in a mouse model of radiation-induced lung fibrosis [188]. Nevertheless, despite successfully completed phase II clinical trials of pamrevlumab in IPF [189], its potential to prevent radiation-induced lung toxicity needs further evaluation.

Apart from cytokines, extracellular adenosine contributes to the development of RILF. It is released by irradiated cells or generated from extracellular adenine nucleotides by the interplay of the ectoenzymes ectoapyrase (CD39) and 5’ectonucleotidase (CD73), respectively [190]. Targeting the CD39/CD73/adenosine axis via administration of PEGylated adenosine deaminase or CD73 antibodies resulted in significantly attenuated RILF in preclinical settings [191] and thus represents a promising pharmacological strategy for future clinical trials.

Several transient receptor potential cation channels (TRPs) are expressed in the lung and have been found to mediate inflammatory and fibrotic processes, such as interstitial edema and lung fibrosis. TRPM2 is involved in acute and late radiation-induced toxicity, and its PARP1-dependent activation upon exposure to ionizing irradiation has been described to contribute to the development of xerostomia in a mouse model [192]. Furthermore, TRPM2-deficient mice exhibit less inflammation and dermatofibrosis in response to radiotherapy as compared to wild type mice [193]. Thus, the role of TRP channels as potential therapeutic target in the prevention of RIP and RILF needs further investigation [194].

Finally, mesenchymal stem cells (MSCs) have been shown to exhibit strong regenerative capacity for several forms of tissue damage [195]. MSCs can successfully migrate towards the injured site in the lung upon irradiation and differentiate into distinct lung cell types, including AT I/II cells and endothelial cells. Preclinical studies reported that lung fibrosis can be modulated by administration of MSCs [195, 196]. In these settings, adoptive transfer of MSCs did limit radiation-induced endothelial cell loss in the early phase after irradiation and promoted tissue repair through the secretion of superoxide dismutase 1 (SOD1) [197] and the anti-fibrotic factors hepatocyte growth factor (HGF) and prostaglandin E2 (PGE2) [198]. Initial phase I trials on IPF confirmed safety of MSC application and reported promising outcomes [199, 200].

### Treatment of RIP and RILF

National and international guidelines recommend treating RIP only if symptomatic with corticosteroids according to clinical severity based on consensus guidelines due to limited clinical data. The treatment should be carried out over at least several weeks and subsequently should be slowly tapered (see Table 4) [201]. Abrupt discontinuation should be avoided in order to prevent early relapse of RIP (rebound phenomenon) with increased severity and higher risk of RILF development. For asymptomatic or subclinical patients, clinical observation without further treatment is recommended. Patients with radiation-induced BOOP usually show fast and effective responses to steroid treatment [202]. Prophylactic administration of antibiotics in RIP can be considered for patients at high risk of bacterial infection, for instance with cancer-associated bronchial stenosis, or for immunocompromised patients. If symptoms persist under treatment with steroids and/or antibiotics, antifungal treatment may be subscribed. Steroid doses can be reduced in combination with azathioprine or cyclosporine A. For individual cases, these agents can be used if corticosteroid treatment fails. Respiratory gymnastics and inhalation of β-sympathomimetics have been reported to be supportive. In severe cases of RIP (CTCAE ≥III), administering oxygen, assisted ventilation and prophylaxis of right heart failure are needed. A successful treatment option for RILF has yet not been established.

**Table 4:** Treatment with corticosteroids in responsive patients with moderate RIP (CTCAE I-II)

Treatment period (days)	Prednisolone dose (mg/day)
1–4	60
5–8	30
9–14	12
> 15 (ca. 6 weeks)	6

## Conclusions

RIP and RILF remain dose limiting forms of radiation-induced lung toxicity with relevant impact on the success of thoracic radiotherapy. Several patient-, disease- and treatment-related factors, namely age, pre-existing lung disease, tumor location, radiation dose, and irradiated volume, need to be considered when trying to predict their risk of occurrence. This is of particular importance in complex settings of multi-modal radio-chemo-immunotherapy with or without prior surgery. Refined radiation delivery techniques, including motion management and treatment verification strategies, can reduce the irradiated lung volume and should be considered for patients with high risk of RIP. The current repertoire of preventive and/or therapeutic intervention by administration of radiation protectors, modifiers, and/or mitigators remains rather limited. But with growing knowledge of the underlying cellular and molecular mechanisms of radiation-induced lung toxicity, promising targets and pathways have been and will be identified to serve as future therapeutic options – preferentially in combination with novel radiation delivery techniques.

## Data Availability

Not applicable.

## Abbreviations

2D: 2-dimensional
3D: 3-dimensional
18F-FDG PET/CT: [18F]fluoro-2-deoxy-2-D-glucose positron emission tomography combined with computed tomography
ACE: Angiotensin-converting enzyme
AT I: Alveolar type I
AT II: Alveolar type II
BOOP: Bronchiolitis obliterans organizing pneumonia
COPD: Chronic obstructive pulmonary disease
CRP: C-reactive protein
CT: Computed tomography
CTCAE: Common terminology criteria for adverse events
CTGF: Connective tissue growth factor
CTV: Clinical target volume
DAMP: Damage-associated molecular pattern
DLCO: Diffusing capacity of lung for carbon monoxide
DNA: Deoxyribonucleic acid
ERK: Extracellular signal–regulated kinase
FEV1: Forced expiratory volume in one second
GTV: Gross tumor volume
HGF: Hepatocyte growth factor
HRCT: High-resolution computed tomography
ICAM-1: Intercellular adhesion molecule 1
ICI: Immune checkpoint inhibition
IGRT: Image-guided radiotherapy
IL: Interleukin
ILA: Interstitial lung abnormality
ILD: Interstitial lung disease
IMRT: Intensity modulated radiotherapy
MAPK: Mitogen-activated protein kinase
MLD: Mean lung dose
MR: Magnetic resonance
MSC: Mesenchymal stem cell
NSCLC: Non-small cell lung cancer
OR: Odds ratio
PAI-1: Plasminogen activator inhibitor-1
PARP1: Poly [ADP-ribose] polymerase 1
PD-1: Programmed cell death 1
PDGF: Platelet-derived growth factor
PD-L1: Programmed cell death 1 ligand 1
PECAM-1: Platelet endothelial cell adhesion molecule 1
PGE2: Prostaglandin E2
PTV: Planning target volume
RILF: Radiation-induced lung fibrosis
RIP: Radiation-induced pneumonitis
ROS: Reactive oxygen species
RTOG: Radiation Therapy Oncology Group
SABR: Stereotactic ablative radiotherapy
SASP: Senescence-associated secretory phenotype
SBRT: Stereotactic body radiotherapy
SMAD: Small mother against decapentaplegic
SOD1: Superoxide dismutase 1
SRF: Serum response factor
TGF-β: Transforming growth factor-β
TIMP: Tissue inhibitor of metalloproteinases
TLR4: Toll-like receptor 4
TNF: Tumor necrosis factor
TRP: Transient receptor potential cation channel
TRPM2: Transient receptor potential cation channel member 2
V20: Lung volume receiving 20 Gy
VMAT: Volumetric modulated arc therapy
α-SMA: α-smooth muscle actin
